# Supporting employees with chronic conditions to stay at work: perspectives of occupational health professionals and organizational representatives

**DOI:** 10.1186/s12889-021-10633-y

**Published:** 2021-03-25

**Authors:** A. R. Bosma, C. R. L. Boot, N. C. Snippen, F. G. Schaafsma, J. R. Anema

**Affiliations:** 1 Department of Public and Occupational Health, Amsterdam UMC, VU University Amsterdam, Amsterdam Public Health Research Institute, Amsterdam, the Netherlands; 2 Department of Health Sciences, Community and Occupational Medicine, University of Groningen, University Medical Center Groningen, Groningen, the Netherlands

**Keywords:** Chronic disease, Occupational health services, Organizations, Prevention, Qualitative research, Work

## Abstract

**Background:**

Supporting employees with chronic conditions can prevent work-related problems and facilitate sustainable employment. Various stakeholders are involved in providing support to these employees. Understanding their current practices and experienced barriers is useful for the development of an organizational-level intervention to improve this support. The aim of this study was to explore the current practices of occupational physicians and organizational representatives, identifying both barriers to providing support and opportunities for improvement.

**Methods:**

Two focus groups with sixteen occupational physicians and seven semi-structured interviews with organizational representatives were held between January and June 2018. Data was analyzed using thematic content analysis.

**Results:**

Several barriers to offer support were identified, including barriers at the organizational level (negative organizational attitudes towards employees with chronic conditions), the employee level (employees’ reluctance to collaborate with employers in dealing with work-related problems), and in the collaboration between occupational physicians and organizational representatives. In addition, barriers in occupational health care were described, e.g. occupational physicians’ lack of visibility and a lack of utilization of occupational physicians’ support. Opportunities to optimize support included a shared responsibility of all stakeholders involved, actively anchoring prevention of work-related problems in policy and practice and a more pronounced role of the health care sector in preventing work-related problems.

**Conclusions:**

Preventing work-related problems for employees with chronic conditions can be achieved by addressing the identified barriers to provide support. In addition, both occupational physicians and organizational representatives should initiate and secure preventive support at the organizational level and in occupational health care. These insights are helpful in developing an intervention aimed at supporting employees with chronic conditions to stay at work.

**Supplementary Information:**

The online version contains supplementary material available at 10.1186/s12889-021-10633-y.

## Background

Having a chronic condition can have a significant impact on one’s working life. Fatigue and physical or cognitive limitations among employees can result in productivity loss, sick-leave, and/or job loss. Staying at work is important for both physical and mental wellbeing and contributes to one’s quality of life [1, 2]. Chronic conditions in the workforce also impact employers. Aside from the financial burden of productivity loss and extended or frequent sick-leave, employers face the practical challenges of securing continuity of skilled personnel and providing employees with needed support and accommodations [3, 4]. With the number of employees with one or more chronic conditions increasing, preventing work-related problems and facilitating sustainable employment for these employees has become more important than ever [4, 5].

Prior research has identified various factors that facilitate sustainable employment for employees with chronic conditions, including work-related, disease-related and personal factors. Our earlier studies among employees with chronic conditions already showed the importance of disclosure and employees expressing their needs to enable them to stay at work [6]. Furthermore, various aspects of the work environment contribute to sustainable employment, such as organizational culture, employee-employer relations, company policies and organizational support [7, 8]. Organizational support and a supportive work environment enable employees with chronic conditions to talk about their condition and ask for support or accommodations if needed, thereby creating the right circumstances for them to stay at work [6, 9]. Therefore, aside from employees with chronic conditions, other stakeholders both within occupational health care and within organizations, can play a role in sustainable employment by providing support to these employees.

Countries vary in how they arrange their occupational health services and which professionals are responsible for this provided care and support to employees with chronic conditions, e.g. occupational health professionals, general practitioners. Even between organizations the way in which occupational health services are organized can differ. In the Netherlands, employers are required to provide occupational health services to their employees, either through an in-house occupational health services department or by having a contract (specifying services and tasks to be performed) with external occupational health services or a self-employed occupational physician (OP). In this context, OPs facilitate sustainable employment by providing employees and employers with support and advice related to work and health [10]. In recent years, the Dutch government has increased the focus on the prevention of work-related problems by adding an amendment to the Occupational Health and Safety legislation which requires organizations to ensure that their employees have the opportunity to access preventive consultation hours with OPs [11]. However, the role of OPs in preventing work-related problems and promoting sustainable work participation remains relatively small, as they are still mostly dealing with employees with existing problems or cases of absenteeism [12, 13].

Aside from OPs, organizational representatives (e.g. management, supervisors, and human resources managers) are relevant stakeholders, as they have an essential role in ensuring organizational support and creating supportive work environments [14]. However, organizational support is not always provided and the support that is offered may not always meet employees’ needs. This may be related to a lack of knowledge or awareness among organizational representatives of the impact of a chronic condition on working life, as shown in a study by Kopnina et al. [15].

Improving support within the work environment could help employees with chronic conditions to stay at work and facilitate sustainable employment. Although many workplace interventions have been developed in the past several years to support these employees, a large proportion of these interventions focus merely on return to work or a reduction in sickness absence. Furthermore, most existing workplace interventions target only individual employees rather than the organization as a whole [16–18]. At present, interventions aimed at the organizational level, directed at *preventing* work-related problems and improving sustainable employment among employees with chronic conditions (i.e. selective or indicated prevention [19]), are scarce [20]. Moreover, implementation of organizational-level interventions is complex due to the involvement of various stakeholders [21].

As we intend to develop an organizational-level intervention aimed at improving support in the work environment for employees with chronic conditions, it is important to first understand the barriers that relevant stakeholders experience when providing support, as well as the opportunities for optimizing this support. Insight in their perspectives can aid in the development of this organizational-level intervention, which could in turn facilitate sustainable employment for employees with chronic conditions. Therefore, the aim of this study is to explore the perspectives of OPs and organizational representatives on current practices and the barriers they face when it comes to providing support and to identify opportunities to improve support for employees with chronic conditions.

## Methods

### Study design

For this study, qualitative research methods were used to capture the perspectives of different stakeholders in the work environment on barriers to and opportunities for improvement of support. Between January and June 2018, focus groups and semi-structured interviews were conducted with OPs and organizational representatives (supervisors and human resources managers). Focus groups were held to explore the perspectives of OPs. Due to time constraints of the organizational representatives, semi-structured interviews were chosen to explore their perspectives. The consolidated criteria for reporting qualitative research (COREQ) were used when designing and reporting the study (Supplementary file 1) [22].

### Recruitment

OPs in the Netherlands regularly meet in continued medical education (CME) groups to discuss cases or topics related to occupational health care. We aimed to include a purposive sample of both self-employed OPs and OPs employed through external occupational health services or within an in-house occupational health services department, of different sizes of organizations. To achieve this, we emailed the chair of two CME groups with a description of the project and a request to use one of their meetings for a focus group session. These CME groups were recruited through the professional network of the researchers and both agreed to participate.

Organizational representatives were recruited through the researchers’ professional and personal network and via snowball sampling, with the intention of including representatives of different sizes of organizations. They were contacted by email with a detailed description of the study and asked to participate in an interview. When participants agreed to participate, a date and time was set at the convenience of the participants. One of the organizational representatives who was contacted was not able to participate in an interview due to time constraints.

### Participants

Two focus groups were held, in which a total of sixteen OPs participated. The first focus group consisted mainly of OPs employed within an in-house occupational health services department of a large organization. In the second focus group, the majority of OPs was self-employed. Self-employed OPs often worked for small- and medium-sized organizations. A total of seven interviews were conducted with organizational representatives of different organizations. Two organizational representatives worked at the same organization. Further characteristics of participants are shown in Table 1.

**Table 1:** Characteristics of focus group and interview participants

Characteristics		Number
Occupation	Occupational physician	16
	Supervisor	3
	Human resources manager	4
Sex	Male	12
	Female	11
Size of participant’s organization	Large (> 500 employees)	16
	Medium (50–500 employees) / Small (< 50 employees)	7

### Data collection

Both focus groups were held at the pre-arranged locations of the CME meetings. Each focus group lasted approximately one and a half hour and was conducted in Dutch. The focus groups were moderated by the primary researcher (AB), a female health scientist with experience in qualitative research. During both focus groups, an observer was present to assist the moderator with monitoring group interaction and taking notes. The focus groups started with some information on the personal background of the researcher. Thereafter, the aim of the study, including the definition of a chronic condition as used in this study (a condition that is continuing or occurring recurrent for a long time and in which there is generally no prospect of full recovery [23]), was explained. This broad definition includes various types of diseases and disorders, both physical and psychological. A script with topics and open questions was developed to aid the moderator and ensure comparability between the focus groups. Topics discussed during the focus groups included: (1) current experiences with supporting employees with chronic conditions and a reflection on an OP’s specific role, (2) barriers to providing support, and (3) potential ways to achieve or create optimal support for employees with chronic conditions. The semi-structured interviews were held at the organizational representatives’ work locations and were also conducted by the primary researcher (AB), with no observer present. Interview duration ranged from 25 to 60 min. The researcher started with introducing herself (in case the participant did not know the researcher prior to the study) and explaining the aim of the study (as described above). For this study, an interview guide with open ended questions was developed to structure the interviews, with topics similar to those in the focus groups (Supplementary file 2). Afterwards, both focus group and interview participants received a gift certificate. As no new themes emerged at the end of data collection, it was concluded that data saturation was achieved. Therefore, no additional focus groups or interviews were conducted.

### Data analysis

The focus groups and interviews were digitally recorded and transcribed verbatim. Summaries of the focus groups, with the main findings on the discussed topics, were made and sent to all participants for member-checking (i.e. to check whether participants agree with or have feedback on the summary made). No feedback or additional comments were received from the focus group participants. With the semi-structured interviews, no member-checking was carried out, as the researcher ended each interview with a small summary of main points mentioned by the organizational representative. Thematic analysis was used to analyze the collected data [24]. The analytical process consisted of several stages, starting with reading and rereading the transcripts. An inductive approach was used to analyze the data, starting with line-by-line coding, thereby using qualitative data indexing software (ATLAS.ti) to assist the coding process. Next, data was searched for similarities and discrepancies, and ultimately grouping and combining codes into subthemes in an iterative manner. The primary researcher (AB) and third co-author (NS) coded all the data. Disagreements in the coding and grouping process were discussed until consensus was reached. The final step, conducted by all researchers in the project team, consisted of clustering the subthemes into main themes. The project team consisted of three health scientists and two OPs. A native English speaker translated representative quotes from the focus groups and interviews, which were added to the text to illustrate the results.

### Ethical considerations

Written informed consent was obtained from all focus group and interview participants. Oral and written information was provided on the confidentiality and anonymity of the results of the study. The Medical Ethics Review Committee of the VU University Medical Center determined that an ethical approval was not required because the Medical Research Involving Human Subjects Act (‘Wet Medisch-wetenschappelijk Onderzoek met mensen’) does not apply to this study.

## Results

The perspectives of OPs and organizational representatives on barriers to provide support and opportunities for improving support were captured in ten themes. An overview of themes and subthemes is presented in Table 2.

**Table 2:** Overview of themes and subthemes

Barriers to provide support	
1. Negative organizational attitudes towards employees with chronic conditions	
• Not wanting to retain employees with chronic conditions and contribute to their sustainable employment	
• Employers’ financial considerations and fear of high costs	
• Employers’ mistrust and co-workers’ jealousy towards needed accommodations	
2. Employees’ reluctance to collaborate with employers in dealing with work-related problems	
• Employees’ non-disclosure of their chronic condition	
• Employees’ lack of cooperation	
3. Lack of skills and knowledge of how to support employees with chronic conditions	
• Employers’ lack of knowledge of rules and regulations	
• Too much medicalization of support	
4. Suboptimal collaboration between OPs and organizational representatives	
• Not meeting each other’s expectations in terms of performance	
• Questioning OPs’ objectivity	
• Impeded communication due to privacy legislation	
5. Lack of utilization of OPs’ support	
• Employers and employees fail to seek preventive support from OPs	
• Employers do not refer employees to preventive consultation hours	
6. OPs’ lack of visibility	
• Employees’ unawareness of the availability of support from OPs	
• The distance between OPs and organizations	
7. OPs’ lack of time and capacity for prevention	
• Too much time is spent on reducing sickness absence rather than on prevention	
• Shortage of OPs	
Opportunities to improve support	
8. Shared responsibility of all stakeholders involved to prevent work-related problems	
9. Actively anchoring prevention of work-related problems in policy and practice	
• Proactive prioritizing prevention in occupational health care	
• Creating a supportive work environment and developing organizational policy	
10. Increasing the role of the health care sector in the prevention of work-related problems	

### Negative organizational attitudes towards employees with chronic conditions

#### Not wanting to retain employees with chronic conditions and contribute to their sustainable employment

Despite OPs’ efforts to educate employers on the added value of employees with chronic conditions, OPs and some organizational representatives described organizations’ unwillingness to support and retain employees with chronic conditions. Instead of offering support, cases were mentioned in which needed work adjustments were not implemented or attempts were made to lay off employees with chronic conditions.

*“And what I also see is that when people are young and they have a medical condition, employers have a tendency of: ‘Well, he still has to work for so many years, so we actually want to get rid of him.’” (Occupational physician)*

Ignorance about the condition or potential solutions to otherwise retain these employees were described as possible causes. In contrast, one organizational representative presented her organization as a ‘social firm’, with employing people with a distance to the labor market as its primary focus.

#### Employers’ financial considerations and fear of high costs

Some organizational representatives spoke of the financial considerations when providing support and the fear of high costs, e.g. for implementing necessary accommodations. Moreover, OPs felt that the Dutch Occupational Health and Safety legislation negatively influenced how organizations support and attempt to retain employees with chronic conditions, by placing a great financial responsibility on employers in case of sickness absence:

*“Employers have to contribute so much financially and for so long, in case employees who have a disability are unable to do their job, so that employers literally select their employees.” (Occupational physician)*

#### Employers’ mistrust and co-workers’ jealousy towards needed accommodations

Several organizational representatives sometimes felt feelings of mistrust towards employees and had doubts about whether accommodations were really needed. Some even felt that employees took advantage of the provided support. In that case, they sought the advice from OPs for confirmation. Moreover, implementation of accommodations could evoke feelings of resentment or jealousy among co-workers, as these might impact their workload (e.g. by transferring tasks to co-workers) or because co-workers would have liked to receive the same accommodations or privileges. This made it more difficult for supervisors to implement accommodations.

### Employees’ reluctance to collaborate with employers in dealing with work-related problems

#### Employees’ non-disclosure of their chronic condition

Employees’ non-disclosure was mentioned by several organizational representatives as an important barrier to provide support, as this complicated communication between them. Some of these organizational representatives emphasized the importance of a relationship build on trust and having sufficient communication skills to bring about disclosure of the chronic condition by the employee, which were not always present.

*“In some cases, everything is out in the open, and the relationship between manager and employee is just fine, so then it is clear. But for many it is not, and that makes communication sometimes difficult.” (Organizational representative)*

In contrast, an organizational representative of a small organization indicated the open workplace culture, where disclosure and expressing needs were fostered. This workplace culture, combined with short lines in communication made it easier for a supervisor to offer support and arrange accommodations, which was echoed by OPs of small organizations.

#### Employees’ lack of cooperation

Several organizational representatives described their difficulties with supporting employees who were not willing to cooperate or to take responsibility for dealing with their chronic condition at work. According to them, this lack of cooperation was the result of various causes, e.g. shame, denial and not accepting how the diagnosis had impacted their work ability. Some organizational representatives mentioned the struggle with getting through to employees and described that they sometimes even felt they needed to impose necessary adjustments on employees (e.g. reduction of working hours). Moreover, some organizational representatives indicated that they perceived a lack of necessary skills to adequately guide employees with the process of acceptance. For one other organizational representative, this lack of cooperation evoked the feeling that the employee did not want to stay at work:

“*Sometimes you have a sort of gut feeling, which has to do with someone not cooperating in making concrete agreements, that someone refuses to take yes, some kind of responsibility, all kinds of clues that made me think: ‘do you really want to stay at work?’” (Organizational representative)*

According to organizational representatives and OPs, actively asking for and being receptive to support were considered crucial in order for them to be able to support employees with chronic conditions and prevent work-related problems.

### Lack of skills and knowledge of how to support employees with chronic conditions

#### Employers’ lack of knowledge of rules and regulations

According to several organizational representatives, difficulties with supporting employees also related to supervisors’ lack of knowledge with regard to laws and regulations that deal with employees’ health. The complexity and changing regulations, and having limited experience in dealing with these laws and regulations were described as underlying causes for this lack of knowledge. Supervisors often turned to their human resources managers whom fulfilled an advisory role in how to comply with the existing laws and regulations.

*“Yes, and I know very little about legislation and regulations, for example. I just rely on a human resources manager. That [legislation and regulations] changes all the time. Yes, now I have had a bit more to do with it, it is quite complex … ” (Organizational representative)*

An organizational representative of a small organization explained the difficulty he had with understanding the complex rules and regulations when starting the company. He considered supporting employees with chronic conditions as a learning process with trial and error.

#### Too much medicalization of support

Several OPs and organizational representatives mentioned struggling with the medicalization of offering support to employees with chronic conditions in the work setting. Although they all wanted to support the employees, they did not want to put too much emphasis on the medical issues and negative consequences of the chronic condition on work, but rather wanted to focus the support on what could be done in the work environment to help these employees.

### Suboptimal collaboration between OPs and organizational representatives

#### Not meeting each other’s expectations in terms of performance

OPs and organizational representatives described several occasions in which their expectations of each other’s functioning were not met, criticizing each other’s performance. Some organizational representatives felt irritated about the defensive or in other cases passive attitude on the part of the OP. Whereas others complained about OPs refraining from any concrete advice, for example after referring an employee for a preventive consultation.

*“You get that in your report: ‘employee is not sick, so can just go to work’. Yes, duh! I knew that. That was not my point.” (Organizational representative)*

Several organizational representatives emphasized the necessity of having clear mutual expectations and for them to clearly communicate their explicit request for advice to OPs.

Meanwhile, some OPs were critical of those at the supervisor level for failures to signal problems or to follow-up on their advice. According to some OPs, the size of the organization also influenced this, highlighting that small organization more often follow-up on OPs’ advice.

*“My experience is that in small companies there is much more cooperation with me and they listen much better [to my advice].” (Occupational physician)*

#### Questioning OPs’ objectivity

Some organizational representatives questioned OPs’ objectivity and pointed out that OPs often take the side of employees. Moreover, they felt that OPs are sometimes too protective of employees. One organizational representative described that she felt that OPs let themselves be persuaded by employees to extent the period on sick leave.

*“In this case, I always contacted the occupational physician in advance if I knew of an upcoming appointment, to see, gosh, what is reasonable [for this employee]? What is possible from a medical point of view? And I noticed that the occupational physician let himself very much, yes, be persuaded by an employee, while I thought is it really all that bad?” (Organizational representative)*

#### Impeded communication due to privacy legislation

Although both OPs and organizational representatives highlighted the importance of clear communication between them, they explained that this was being complicated with the renewed European privacy legislation (General Data Protection Regulation). This new law prohibited OPs from discussing the details surrounding the condition of the employee with organizational representatives.

### Lack of utilization of OPs’ support

#### Employers and employees fail to seek preventive support from OPs

OPs explained that employees with chronic conditions often visit them only after problems have arisen, making it more difficult to provide preventive support. According to some OPs and organizational representatives, most employees want to continue their work as much as possible and manage their situation themselves, instead of asking an OP for support. As a result, OPs were often not aware of the number of employees with chronic conditions in the organization, and therefore the extent of the problem. Moreover, several OPs indicated that employers or supervisors too often try to solve work-related health problems themselves, instead of seeking the advice from their OP:

*“As a self-employed OP, I work for several smaller organizations, many of which using their own ‘self-management model’. Since then [the use of ‘self-management models’ in organizations], there are a thousand doctors on the work floor. Employees no longer have to go to the occupational physician, because they [supervisors] know everything about Parkinson's disease and diabetes.” (Occupational physician)*

#### Employers do not refer employees to preventive consultation hours

Organizational representatives indicated that they only occasionally refer employees to a preventive consultation with their OP in order to obtain advice on preventing work-related problems in the future. One representative pointed out to experience a feeling of taboo around preventively referring employees to the OP within their organization. In addition, the organizational representative of a small organization explained that he had never thought about the possibility of preventively referring an employee to their OP.

*“I have never really thought about it [preventively referring employees]. [ … ] But I think yes, that would certainly, in view of prevention on the long term, be a wise thing to do.” (Organizational representative)*

On the other hand, the organizational representative of the ‘social firm’ explained that employees are clearly informed about the possibility of preventively consulting the OP and that their preventive consultation hours were widely used.

### OPs’ lack of visibility

#### Employees’ unawareness of the availability of support from OPs

OPs talked about their lack of visibility to both employers and employees, which in turn negatively impacted their accessibility. Some OPs described that employees are not always aware of the existence of an OP or the possibility to consult an OP, as illustrated by the quote below. In addition, OPs described that many employees persist in their idea that OPs are only available for sickness absence consultations.

*“With a larger organization, you are more like a mountain [clearly visible], but with many smaller organizations, employees say: ‘oh, I didn't know we had an occupational physician at all.’ Yes, then they get sick and get called in by me, only then do they know … ” (Occupational physician)*

#### The distance between OPs and organizations

Some organizational representatives spoke of the psychological and physical distance they felt between them and their OP. One organizational representative expressed that the psychological distance he felt to the OP from the external occupational health service, negatively influenced accessibility of their OP:

*“No, I have to be honest, I do not even know the name of our occupational physician … ” (Organizational representative)*

While on the other hand, another organizational representative spoke of the ideal situation of their OP’s weekly consultation hours at the workplace. Also OPs mentioned the distance between organizations and OPs; a greater physical distance made it harder for them to provide adequate support to employers and employees. For some of them this was even a reason for not wanting to work through a case management agency anymore. In order to reduce the threshold for employers and employees to seek support from them, several OPs mentioned making regular visits to the workplace.

### OPs’ lack of time and capacity for prevention

#### Too much time is spent on reducing sickness absence rather than on prevention

According to OPs and organizational representatives, current legislation has pushed OPs more towards dealing with sickness absence, as social security is getting stripped further and further, making it more difficult for employees to receive benefits (e.g. disability benefits). Moreover, OPs described making agreements with organizations about the number of hours they work and the tasks they should perform, with organizations often demanding to focus mostly on absenteeism. Several OPs explained that as a result, they spend the majority of their working hours on reducing sickness absence, leaving less time available for preventing work-related problems and preventive support.

*“Well, we now have such a nice new amendment of the labor legislation, which states that occupational physicians must be provided with more time for prevention. But when I look at my clients [organizations], they want to [focus on prevention] … but in the end there is also a limit to my agenda. You have agreed to one day a week [number of days working for the organization], but if absenteeism increases rapidly, then that is what you focus on.” (Occupational physician)*

#### Shortage of OPs

In addition, some OPs and organizational representatives spoke of the current shortage of available OP capacity. One representative described the difficulty of finding a new OP after their current OP gave notice of his resignation. Some OPs also described the problem of there being a shortage of OPs, as indicated by the many job offers.

*“But yes, that also applies to our occupational group... shortage. I am approached several times a week, eh, for cooperation, if I want to do a job. Then I wonder, you know? That is a problem.” (Occupational physician)*

### Shared responsibility of all stakeholders involved to prevent work-related problems

Both OPs and organizational representatives stated that it is everyone’s social duty to keep employees with chronic conditions at work. Preventing work-related problems and facilitating sustainable employment requires a joint effort and shared responsibility of all stakeholders involved (i.e. stakeholders in organizations, including employees and in occupational health care).

*“I sometimes say to a manager: ‘It is simply a social obligation that we have, to retain the people with a chronic condition as well. That you have a diverse team. Yes, you also have an exemplary role, if there is a problem, we will solve it. No nagging about that.’” (Occupational physician)*

### Actively anchoring prevention of work-related problems in policy and practice

#### Proactive prioritizing prevention in occupational health care

Both OPs and organizational representatives emphasized that OPs have to be more proactive in taking up more preventive tasks and motivating organizations to focus more on prevention instead of reducing sickness absence. This requires OPs to make their role clear to supervisors, human resources managers, and employees, and to show to the organization their added value in preventing work-related problems. OPs indicated that, to ensure the embedding of prevention in occupational health care, they have to practice what they preach and negotiate the allocation of preventive tasks in their contracts.

*“As an occupational group, we are simply too much driven by that whole absenteeism and uhm, we just have to have the guts to say: ‘well, and from now on there are no extra absenteeism consultation hours, but instead more consultations about prevention.’” (Occupational physician)*

Moreover, several organizational representatives pointed out that occupational health services could also play a more pronounced role in proactively promoting preventive support by addressing the importance of prevention, taking preventive measures, and guiding and educating organizations on how to support employees with chronic conditions.

#### Creating a supportive work environment and developing organizational policy

According to OPs, as well as organizational representatives, an organization should ensure a work environment in which employees feel supported by their organization. Furthermore, there should be a clear organizational policy that illustrates an organization’s view on preventing work-related problems among employees with chronic conditions, and that facilitates the implementation of accommodations and preventive support.

*“Actually, it would be very nice if this is in the mission statement of a company: ‘for people who are chronically ill, our goal is to let people work optimally for as much as possible, for example. And we do this and this and this [to accomplish the mission statement] and that is what we're training our executives for’ you know. That's a really nice idea I think, if that's clear. Yes, so that … look, if it [working with a chronic condition] is not an issue you don't need that information at all, but at the same time it is also nice to work in such an organization, where it is just transparent.” (Organizational representative)*

An OP indicated that a change in work culture within an organization is sometimes required to achieve such a supportive work environment.

### Increasing the role of the health care sector in the prevention of work-related problems

OPs stressed the important role of general practitioners, medical specialists, and specialized nurses in preventing work-related problems for employees with chronic conditions. Although in the Netherlands, the health care sector is not responsible for occupational health care, OPs described several aspects within the broader health care system that could improve the prevention of work-related problems. First, more attention on employment and paid work in the course of treatment. Second, if health care professionals would refer people with a (newly diagnosed) chronic condition to the OP more often, it would enable OPs to offer support and advice on preventing work-related problems at an earlier stage. Third, OPs indicated that a good collaboration between themselves and health care professionals is essential for providing adequate support and the implementation of accommodations which are fitted to the needs of employees with chronic conditions.

*“What you see is that employment is gradually coming into those guidelines [of medical specialists], but it is not yet in the minds of all specialists and care providers in healthcare. That's one thing. And they don't think in terms of functioning, like a rehabilitation doctor does or we do [ … ]. And that, this other way of thinking, that is what I really miss the most.” (Occupational physician)*

## Discussion

This study described the experiences and perspectives of OPs and organizational representatives on barriers to provide support and opportunities to improve support for employees with chronic conditions in order to prevent work-related problems and facilitate sustainable employment. OPs and organizational representatives identified various barriers for providing support, including negative organizational attitudes towards employees with chronic conditions, employees’ reluctance to collaborate with employers in dealing with work-related problems, OPs’ lack of visibility and a lack of utilization of OPs’ support. OPs and organizational representatives also identified opportunities for improving preventive support and sustainable employment for employees with chronic conditions. Opportunities included a shared responsibility of all stakeholders involved for preventing work-related problems, actively anchoring prevention of work-related problems in policy and practice and increasing the role of the health care sector in the prevention of work-related problems.

### Comparison to the literature

Our study identified several barriers to provide support in the work environment, e.g. negative organizational attitudes towards employees with chronic conditions, employees’ non-disclosure or employers’ lack of knowledge of the rules and regulations, which are in line with other studies [25–28]. In view of negative organizational attitudes towards employees with chronic conditions, financial considerations were of importance. In the Netherlands, employers can apply for financial compensation and premiums to reduce costs for the support of employees with chronic conditions. However, little use is made of these possibilities, because of a lack of knowledge of their availability and the complexity of the terms [29]. When comparing our findings to the perspectives of employees with chronic conditions, two qualitative syntheses show us that these employees often struggle with prejudice, judgement and mistrust in the work environment, and that employees try to avoid a negative image, which relates to the negative organizational attitudes we found in this study [30, 31]. Employees’ non-disclosure and a lack of cooperation hampered the offering of support by organizational representatives. This correlates to two of our earlier studies among employees, which identified disclosure as an important facilitator for staying at work. However, whether employees disclose their chronic condition is very much dependent on the context, being more likely to disclose in a supportive work environment [6, 9]. This endorses the need for creating supportive work environments as found in this study, which is in line with other studies [32–34]. A more pronounced role of the health care sector in preventing work-related problems, as identified in this study, was also mentioned by employees with chronic conditions, and reflects the importance of making work an essential part in the course of treatment [6, 31].

Also barriers in occupational health care were found, such as a lack of use of OPs’ support. The desire of employees and employers to solve problems on their own, was seen as one of the reasons for this. However, our study among employees also showed dissatisfaction with support offered by OPs, which could also be a contributing factor, as this kept employees from seeking additional support from OPs [6]. We also found that some organizational representatives appeared ambivalent to refer employees preventively to OPs, which is in line with a study by Paulsson et al., that also showed a lack of use of suggested expertise of occupational health professionals [35]. Moreover, we showed that OPs still spend most of their time on reducing sickness absence, as agreed upon in their contracts with employers. This implies that occupational health care currently revolves around reactive interventions instead of a proactive preventive approach, which was also found in other studies [35, 36]. Although the European Union sets basic rules for arranging occupational health services, countries differ in how occupational health services are implemented in their national legislation [37]. For Dutch OPs, additional occupational guidelines are available to improve the quality of care. However, these guidelines focus mostly on return to work instead of preventing work-related problems. Moreover, these guidelines are not widely used, due to OPs doubts about usefulness and feasibility in practice [38]. Our study clearly demonstrated that despite obligated by law and aided by guidelines, it is difficult for OPs to use their full potential in light of preventive tasks and promoting selective and indicated prevention in organizations and in occupational health care.

The suboptimal collaboration between OPs and organizational representatives is another important finding, e.g. organizational representatives’ feelings of OPs being on the side of employees. In contrast to employers, many employees with chronic conditions have the impression that OPs mostly represent the interest of the employer [6]. This shows the difficult position OPs are in, as they ought to be independent advisors, hired by employers and representing the interests of employees at the same time. Good collaboration and communication between employers and OPs can optimize service provision. A systematic review by Halonen [39] pointed out the importance of a clear set of services with the flexibility to adjust these to organizational needs, a long-term collaboration, trust, frequent contact, and a shared goal between employers and occupational health services providers [39]. Moreover, expressing mutual expectations and evaluating offered services adds to the quality of the collaboration [40].

### Strengths and limitations

This study illustrated the barriers that OPs and organizational representatives face when it comes to providing support, as well as potential opportunities for improving support for employees with chronic conditions. This study showed the broad perspectives of different types of OPs and of organizational representatives, working for various organizations, using the strengths of two different types of qualitative research methods. Focus groups provided us with a broad insight into OPs’ perspectives, whereas interviews allowed us to gain in-depth understanding of the relevance of the particular organizational context. The findings provide valuable input for the development of an organizational-level intervention for improving support for employees with chronic conditions.

However, limitations for this study can also be identified. First, of the approximately 140 CME groups in the Netherlands, we only used two CME groups for a focus group session. Nonetheless, in this explorative study, representatives of different types of OPs (e.g. self-employed vs. employed at an in-house occupational health services department) were present in one or both of these two groups. Second, a relatively small number of OPs and organizational representatives worked for small- and medium-sized organizations. Although including more participants from small- or medium-sized organizations would perhaps have yielded additional findings, we believe that our heterogeneous group of participants identified the most important barriers and opportunities to improve support. A third limitation is that the results describe the experiences and perspective of OPs and organizational representatives solely in the Dutch context. However, although some findings might only apply to the Dutch situation and are difficult to translate to other countries, many findings are also internationally relevant and of value for other countries, e.g. the lack of use of occupational health professionals’ expertise and the need for anchoring prevention in an organizational policy. Furthermore, this study showed that making occupational health services mandatory in legislation, does not always have the desired effect.

### Practical implications

Preventing work-related problems by providing preventive support can facilitate sustainable employment for employees with chronic conditions, as well as lower employers’ financial burden due to sickness absence. Moving towards more selective or indicated prevention requires changes within organizations as well as occupational health care. Based on our findings, several recommendations can be made on how to improve preventive support for employees with chronic conditions.

In general, organizations must pursue a more proactive and preventive approach, focusing more on preventing work-related problems of employees with chronic conditions (i.e. selective or indicated prevention) rather than on reducing sickness absence. Current legislation has shown to be insufficient for promoting prevention. However, as the amendment of the Occupational Health and Safety legislation (i.e. with more focus on prevention) is relatively new, a clear effect may become visible in the near future. More extensively enforcing compliance to this legislation can however be helpful for achieving the change to a preventive approach. In addition, other ways must be sought to move organizations towards the preventive approach. As for many organizations financial consideration are important, the economic benefits of prevention and preventive support must be made clear. For many supportive activities, it is not always immediately clear whether the costs outweigh the benefits [41]. The benefits of providing high quality preventive support to employees with chronic conditions could lie in generating increased employee motivation and satisfaction and a better corporate image, as also seen in the prevention of workplace accidents and occupational illnesses [42]. Organizations’ awareness of the benefits of preventing work-related problems and preventive support would make it more feasible for OPs to expand their preventive duties.

Making the prevention of work-related problems a shared responsibility of all stakeholders involved, is crucial for improving sustainable employment of employees with chronic conditions. A study by Philips et al. emphasized the importance of the commitment of upper management to retaining these employees [27]. Furthermore, OPs or other occupational health professionals could work more closely together with organizations, increasing their visibility, and together develop an organizational policy aimed at preventing work-related problems, tailored to the specific needs of organizations. A study by Schmidt et al. even described that an effective and strategic collaboration between occupational health professionals and organizations led to a shift towards a more preventive approach of utilizing occupational health services [43]. To tackle the shortage of OPs, the intake in the training program to become an OP must increase, by making the profession of occupational physician more attractive for young doctors [44]. Moreover, in-house and external occupational health services can contribute to the prevention of work-related problems by promoting preventive actions within organizations, for example by educating employers on the importance and potential benefits of prevention. Finally, health care professionals must be educated on the importance of integrating work in the course of treatment and the possibility of (preventively) referring patients to OPs. Joint educational programs can be used to improve this inter-disciplinary collaboration [45].

Although disease-related factors and personal factors also play a role in sustainable employment, there is much to be gained by addressing work-related factors. Prevention in organizations and occupational health care remains difficult, despite the available expertise of OPs on work and health [46]. We will therefore use the results of this study for the development of an organizational-level intervention aimed at improving support in the work environment for employees with chronic conditions. By making OPs an essential part of this organizational-change intervention, their visibility will improve. This could put them in a better position to perform their preventive tasks and collaborate closely with all relevant stakeholders in the organizations to create supportive work environments and prevent work-related problems for employees with chronic conditions.

### Research recommendations

This study was a first step in providing insight into the preventive support offered by OPs and organizational representatives. However, more research should be conducted on the economic benefits of preventing work-related problems among employees with chronic conditions (selective and indicated prevention) and how prioritizing prevention over absenteeism could be promoted within organizations. Our findings provided input for the development of an organizational-level intervention to improve support. Subsequently, the implementation process and effectiveness of such an intervention must be explored. As organizations differ in their size, structure, and other organizational factors, the implementation process and effectiveness of the intervention that will be created should be investigated in various types of organizations.

## Conclusion

This study showed the perspectives of OPs and organizational representatives on the barriers for providing support and opportunities to improve preventive support for employees with chronic conditions. Barriers were identified at the organizational level (negative organizational attitudes towards employees with chronic conditions), the employee level (employees’ reluctance to collaborate with employers in dealing with work-related problems) and in the collaboration between OPs and organizational representatives. In addition, barriers in occupational health care were described, e.g. a lack of OPs’ visibility and a lack of utilization of OPs’ support. Shared responsibility of all stakeholders involved, actively anchoring prevention of work-related problems in policy and practice and a more pronounced role of the health care sector in preventing work-related problems can optimize preventive support and facilitate sustainable employment for employees with chronic conditions.

## Supplementary Information

**Additional file 1: Supplementary file 1.** COREQ checklist - The completed COREQ (32-item) checklist

**Additional file 2: Supplementary file 2.** Interview guide - The interview guide used for the interviews with organizational representatives

## Data Availability

The datasets generated and/or analyzed during the current study are not publicly available due to identifying information, but are available from the corresponding author on reasonable request.

## Abbreviations

CME: Continued medical education
OP: Occupational physician
