# Supplementary material for: Supporting employees with chronic conditions to stay at work: perspectives of occupational health professionals and organizational representatives
Source: BMC Public Health. 2021 Mar 25;21:592. doi: 10.1186/s12889-021-10633-y (PMC7992826; doi:10.1186/s12889-021-10633-y)
Supplement: Supplementary file 1 — Additional file 1: Supplementary file 1. COREQ checklist - The completed COREQ (32-item) checklist [file 12889_2021_10633_MOESM1_ESM.pdf]

Consolidated criteria for reporting qualitative studies (COREQ): 32-item checklist

**No**	**Item**	**Guide questions/description**	
**Domain 1: Research team and reflexivity**			
Personal Characteristics			
1.	Interviewer/facilitator	Which author/s conducted the interview or focus group?	Methods, paragraph “data collection”
2.	Credentials	What were the researcher's credentials? *E.g. PhD, MD*	MSc, Title page
3.	Occupation	What was their occupation at the time of the study?	Methods, paragraph “data collection”
4.	Gender	Was the researcher male or female?	Methods, paragraph “data collection”
5.	Experience and training	What experience or training did the researcher have?	Methods, paragraph “data collection”
Relationship with participants			

No	Item	Guide questions/description	
6.	Relationship established	Was a relationship established prior to study commencement?	Methods, paragraph “recruitment”
7.	Participant knowledge of the interviewer	What did the participants know about the researcher? e.g. *personal goals, reasons for doing the research*	Methods, paragraph “data collection”
8.	Interviewer characteristics	What characteristics were reported about the interviewer/facilitator? e.g. *Bias, assumptions, reasons and interests in the research topic*	Discussion, paragraph “strengths and limitations”
**Domain 2: study design**			
Theoretical framework			
9.	Methodological orientation and Theory	What methodological orientation was stated to underpin the study? *e.g. grounded theory, discourse analysis, ethnography, phenomenology, content analysis*	Methods, paragraph “data analysis”
Participant selection			
10.	Sampling	How were participants selected? *e.g. purposive, convenience, consecutive, snowball*	Methods, paragraph “recruitment”

No	Item	Guide questions/description	
11.	Method of approach	How were participants approached? e.*g. face-to-face, telephone, mail, email*	Methods, paragraph “recruitment”
12.	Sample size	How many participants were in the study?	Methods, paragraph “participants”
13.	Non-participation	How many people refused to participate or dropped out? Reasons?	Methods, paragraph “recruitment”
Setting			
14.	Setting of data collection	Where was the data collected? e.*g. home, clinic, workplace*	Methods, paragraph “data collection”
15.	Presence of non-participants	Was anyone else present besides the participants and researchers?	Methods, paragraph “data collection”
16.	Description of sample	What are the important characteristics of the sample? *e.g. demographic data, date*	Methods, paragraph “participants”
Data collection			
17.	Interview guide	Were questions, prompts, guides provided by the authors? Was it pilot tested?	Methods, paragraph “data collection”

No	Item	Guide questions/description	
18.	Repeat interviews	Were repeat interviews carried out? If yes, how many?	N/A
19.	Audio/visual recording	Did the research use audio or visual recording to collect the data?	Methods, paragraph "data analysis"
20.	Field notes	Were field notes made during and/or after the interview or focus group?	Methods, paragraph "data collection"
21.	Duration	What was the duration of the interviews or focus group?	Methods, paragraph "data collection"
22.	Data saturation	Was data saturation discussed?	Methods, paragraph "data collection"
23.	Transcripts returned	Were transcripts returned to participants for comment and/or correction?	No, only summaries. Methods, paragraph "data analysis"
**Domain 3: analysis and findings**z			
Data analysis			

No	Item	Guide questions/description	
24.	Number of data coders	How many data coders coded the data?	Methods, paragraph “data analysis”
25.	Description of the coding tree	Did authors provide a description of the coding tree?	Results, table 2
26.	Derivation of themes	Were themes identified in advance or derived from the data?	Methods, paragraph “data analysis”
27.	Software	What software, if applicable, was used to manage the data?	Methods, paragraph “data analysis”
28.	Participant checking	Did participants provide feedback on the findings?	Methods, paragraph “data analysis”
Reporting			
29.	Quotations presented	Were participant quotations presented to illustrate the themes / findings? Was each quotation identified? e.*g. participant number*	Results section
30.	Data and findings consistent	Was there consistency between the data presented and the findings?	Yes

No	Item	Guide questions/description	
31.	Clarity of major themes	Were major themes clearly presented in the findings?	Results, paragraph 1 and table 2
32.	Clarity of minor themes	Is there a description of diverse cases or discussion of minor themes?	Results section
