# Supplementary material for: Supporting employees with chronic conditions to stay at work: perspectives of occupational health professionals and organizational representatives
Source: BMC Public Health. 2021 Mar 25;21:592. doi: 10.1186/s12889-021-10633-y (PMC7992826; doi:10.1186/s12889-021-10633-y)
Supplement: Supplementary file 2 — Additional file 2: Supplementary file 2. Interview guide - The interview guide used for the interviews with organizational representatives [file 12889_2021_10633_MOESM2_ESM.pdf]

Interview guide - Organizational Representatives

- Are you aware of any employees having a chronic condition within the organization?
- How do you reflect on your role regarding (preventively) supporting employees with chronic conditions? What tasks do you see for yourself?
- How do you currently support employees with chronic conditions in their work?
    - Accommodations? Training? Other support?
    - Did the occupational physician play a role in this?
    - Do you refer employees preventively to the occupational physician? Do you have any idea if this helps them?
    - How is your collaboration with the occupational physician?
        - Do you have contact with the occupational physician about employees? Even if they are not (yet) on sick leave?
        - What does that contact consist of? Who initiates this?
        - Could collaboration be improved? If yes, how could this be improved?
- What barriers do you encounter when supporting employees with chronic conditions?
- You want to take the best possible care of your employees. How could (preventive) support for employees with chronic conditions be improved? What could help you with improving (preventive) support to these employees?
